# Transcriptional profiling upon T cell stimulation reveals down-regulation of inflammatory pathways in T and B cells in SLE versus Sjögren’s syndrome

**DOI:** 10.1038/s41540-023-00319-z

**Published:** 2023-12-15

**Authors:** Gino Kwon, Annika Wiedemann, Lisa M. Steinheuer, Ana-Luisa Stefanski, Franziska Szelinski, Tomas Racek, Andreas Philipp Frei, Klas Hatje, Tony Kam-Thong, David Schubert, Thomas Schindler, Thomas Dörner, Kevin Thurley

**Affiliations:** 1 Systems Biology of Inflammation, German Rheumatism Research Center, a Leibniz-Institute, Berlin, Germany; 2 Rheumatology and Clinical Immunology, Department of Medicine, Charité-Universitätsmedizin Berlin, Berlin, Germany; 3 Biomathematics Division, Institute of Experimental Oncology, University Hospital Bonn, Bonn, Germany; 4 Roche Pharma Research and Early Development, Pharmaceutical Sciences, Roche Innovation Center Basel, F. Hoffmann-La Roche Ltd, Basel, Switzerland; 5 Roche Pharma Research and Early Development, Immunology, Infectious Diseases and Ophthalmology (I2O) Discovery and Translational Area, Roche Innovation Center Basel, F. Hoffmann-La Roche Ltd, Basel, Switzerland; 6 Product Development Immunology, F. Hoffmann-La Roche AG, Basel, Switzerland

**Keywords:** Computational biology and bioinformatics, Rheumatology

## Abstract

Systemic lupus erythematosus (SLE) and primary Sjögren’s syndrome (pSS) share clinical as well as pathogenic similarities. Although previous studies suggest various abnormalities in different immune cell compartments, dedicated cell-type specific transcriptomic signatures are often masked by patient heterogeneity. Here, we performed transcriptional profiling of isolated CD4, CD8, CD16 and CD19 lymphocytes from pSS and SLE patients upon T cell stimulation, in addition to a steady-state condition directly after blood drawing, in total comprising 581 sequencing samples. T cell stimulation, which induced a pronounced inflammatory response in all four cell types, gave rise to substantial re-modulation of lymphocyte subsets in the two autoimmune diseases compared to healthy controls, far exceeding the transcriptomic differences detected at steady-state. In particular, we detected cell-type and disease-specific down-regulation of a range of pro-inflammatory cytokine and chemokine pathways. Such differences between SLE and pSS patients are instrumental for selective immune targeting by future therapies.

## Introduction

Systemic lupus erythematosus (SLE) and primary Sjögren’s syndrome (pSS) are systemic autoimmune diseases that share clinical, immunological, and genetic features but present also disease-specific traits. Our understanding of the clinical and immunological etiology remains incomplete, but it is well established that dysregulation of innate and adaptive immune responses leading to breach of self-tolerance, expansion of auto-reactive B and T lymphocytes, production of autoantibodies and a continuous secretion of pro-inflammatory cytokines characterize pathologic features of SLE and pSS^1–3^. Both diseases are considered complex conditions in which genetic predisposition, environmental trigger and epigenetic mechanisms contribute to disease induction and maintenance. Even though B lineage cells are known to be key components of the pathophysiology of these diseases^4,5^ no profound knowledge is available about their cross-talk with other lymphocytes subsets like NK cells, CD4+ /CD8+ T cells in SLE and pSS.

The impact of genetic susceptibility on the development of SLE and pSS has been well demonstrated in a number of large-scale genome-wide association studies^6–9^, uncovering a large fraction of genetic heritability. A substantial number of major genetic susceptibility loci are shared between both diseases, such as variants of *HLA class II* and upregulation of interferon IFN regulated genes like *IRF5* and *STAT4*, describing another hallmark of both diseases, referred to as IFN signature^9–14^. In addition, certain risk alleles, i.e., FcR genetic variants, are considered to explain B cell hyperactivation. More recently, both bulk and single-cell transcriptomic sequencing studies have identified additional distinctive blood-transcriptional signatures associated with myeloid inflammation and B cell-related pathways, and have revealed huge heterogeneity also at the single-cell level, for both SLE and pSS^15–20^. In particular, SLE and pSS patients exhibit changes in the abundance and expression of IFN signature genes in various populations of the lymphocyte and myeloid cell compartments^15,19^. However, those studies rely on analyses of unstimulated peripheral blood mononuclear cells (PBMC). Transcriptomic data sets containing sorted immune cell populations are available for SLE and under unstimulated steady-state conditions only^21,22^.

Here, following the hypothesis that transcriptomic differences in autoimmune disease may be more pronounced under conditions of tonic immune stimulation such as in an acute flare of the disease, we assessed gene-expression profiles from distinct sorted populations of B cells, NK cells, and CD4+ and CD8+ T cells at steady-state and upon 18 h anti-CD3 priming of PBMC. Using that approach, we identified a broad range of cell-type and disease-specific molecular differences in the expression profiles compared to healthy human donors, culminating in a modulation of cytokine and cell-cell communication signatures towards anti-inflammatory pathways in SLE but not in pSS.

## Results

### Transcriptional profiling of sorted cell populations reveals cell-type specific gene expression changes in autoimmune disease

**Fig. 1:**
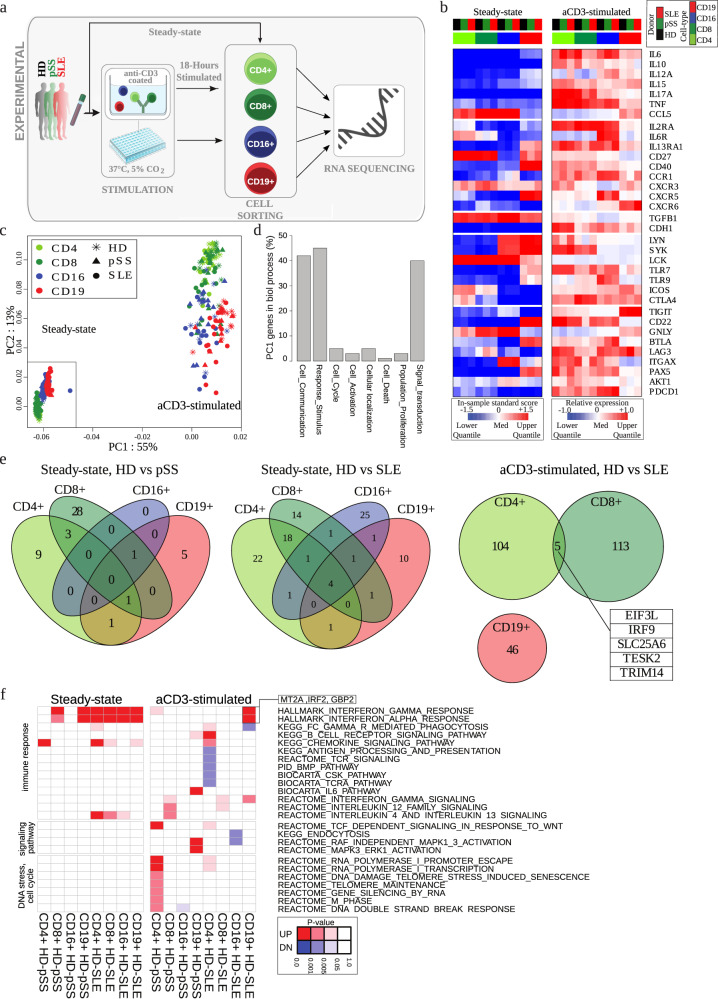
Transcriptional profiling of cell-type specific gene signatures in SLE and primary Sjögren’s patients versus healthy controls. **a** Overview of the study. Flow-sorted individual cell samples were subjected to RNA-sequencing at steady-state or after 18 h of cell culturing with/out anti-CD3 stimulation. In total, we obtained RNA-sequencing data from 581 samples, which were derived from 72 donors of three donor groups (healthy donors, pSS, SLE) and four cell types (cf. Supplementary Fig. 1a). **b** Expression heatmap of selected signature genes of autoimmune disease across all donor groups and cell types. Shown are normalized expression values (steady-state) or relative expression values as compared to control (stimulated), which were averaged across donors for each condition. **c**, **d** Principal component analysis. **c** Symbols represent different donor groups and colors different cell types, as indicated, **d** overlap of PC1 genes with indicated gene ontology terms, see Supplementary Table 1 for details. **e** Venn diagrams of differentially expressed genes (DEG) of indicated donor group comparisons. **f** Pathway over-representation analysis. Instead of the DEG shown in panel D, the top 50 up- and down-regulated genes by false-discovery rate (Supplementary Table 1) were used for the analysis in each condition. *P*-values were calculated using a hypergemetric test and the full list of enriched pathways is provided in Supplementary Table 1.

We selected 25 SLE and 23 pSS patients along with 24 healthy donors, according to pre-specified inclusion criteria and disease classification criteria^23,24^ (Table 1). In particular, we only selected patients with established diagnosis that did not receive high-dose corticosteroids at the time of the study (median dose 5 mg/d). Peripheral blood samples from each donor were sorted for CD4+ (Th cells) and CD8+ (CTL) T lymphocytes, CD16 + CD7+ cells (NK cells), and CD19+ cells (B cells) (Supplementary Fig. 1), and were subjected to RNA-sequencing (Fig. 1a, “steady-state”). A large part of the blood samples was also analyzed after ex vivo incubation in the presence or absence of T cell stimulation by anti-CD3 (aCD3) for 18 h, resulting in a data set comprising 581 sequencing samples in total (Fig. 1a, “stimulation”, and Supplementary Fig. 2a). In pre-experiments, we determined 18 h to be the optimal time point where the cells are activated, but do not proliferate yet (data not shown). Since in autoimmune patients, cells are thought to be pre-activated, we did not add co-stimulatory agonists (CD28) to the culture but rather expected the presence of co-stimulation by means of accompanying cell types. As expected, T cell stimulation induced up-regulation of inflammatory genes and down-regulation of anti-inflammatory genes in T cells, including cytokines and related signaling pathways (SYK, LYN, CD40, LCK, STAT1, AKT1), chemokine receptors and checkpoint molecules, such as PDCD1, BTLA, LAG3, TIGIT, ICOS, ITGAX, and CTLA4 (Fig. 1b, Supplementary Fig. 2b). Intriguingly, gene-expression changes were not limited to CD3 positive T cells only but also found in B and NK cells.

**Table 1:** Donor demographics and inclusion criteria.

**Patient demographics**	**pSS**	**SLE**	**Healthy donors**
Group size (*n*)	23	25	24
Age (y, mean ± SD)	50 ± 12	40 ± 13	34 ± 11
Female % (n)	96 (22)	92 (23)	83 (20)
Current disease activity (mean ± SD)	ESSDAI 3.6 ± 3.5	SLEDAI 4.0 ± 3.3	n.a.
**Inclusion criteria**	**pSS**	**SLE**	
1.	18–75 y female/male	18–65 y female/male	
2.	Established diagnosis of pSS as defined by consensus critera for the classification of pSS according to the 2016 revised ACR/EULAR classification criteria	Established diagnosis of SLE as defined by the 1997 update of the 1982 ACR Revised Criteria of SLE	
3.	Positive anti-nuclear antibodies (ANA) > 1:160 and positive anti-Ro/SS-A	Positive anti-nuclear antibodies (ANA) > 1:160 and/or positive anti-dsDNA antibodies	
4.	At least one of the following criteria: anti-La/SS-B, hypergammaglobulinemia > 15 g/L, increased levels of serum immune-globulin, free light chains, biopsy proven, activity in at least one domain	History of SLE symptoms ≥ 6 months	
5.	Oral corticosteroids ≤ 7.5 mg with stable dose of prednisone or equivalent over 4 weeks	Oral corticosteroids ≤ 15 mg of prednisone or equivalent with stable dose during the study	
6.	Hydroxychloroquine, chloroquine, quina-crine, methotrexate, leflunomide, azathio-prine, mycophenolate or sulfasalazine doses table for at least 4 weeks prior to screening	Hydroxychloroquine, chloroquine, quinacrine, methotrexate, leflunomide, azathioprine, mycophenolate, or sulfasalazine doses table for at least 4 weeks prior to screening	
**Medication**	**pSS**	**SLE**	
w/o systemic therapy % (n)	39 (9)	12 (3)	
Prednisolone % (n)	17 (4)	76 (19)	
median dose (mg/d) [IQR]	5 [5;5.25]	5 [3.25;5]	
Hydroxychloroquine % (n)	48 (11)	56 (14)	
Mycophenolate mofetil % (n)	—	24 (6)	
Azathioprine % (n)	—	32 (8)	
Methotrexate % (n)	—	16 (5)	
Belimumab % (n)	—	12 (3)

To derive a first overview regarding overall gene expression patterns before and after T cell stimulation, we performed principal component analysis (PCA) and correlation analysis (Fig. 1c, Supplementary Fig. 2c, d, Supplementary Table 1). Under steady-state conditions, the rather subtle disease-specific differences (correlation coefficient r > 0.88 throughout, Supplementary Fig. 2d) were largely masked by heterogeneity among individual donors, indicated by low variability in the PCA (Fig. 1c, Supplementary Fig. 2c). After T cell stimulation, sample-to-sample differences were amplified in general (r < 0.6 in many cases, Supplementary Fig. 2d), and notable differences between donor groups became apparent in the PCA plot, for instance in the case of B cells compared between healthy donor and SLE samples (Fig. 1c). That pattern of an increase in both overall sample dissimilarity and disease-specific differences after T cell stimulation was also reflected on the level of individual immune-cell related genes (Fig. 1b and Supplementary Fig. 2b). Notably, T cell stimulation induced a systematic change in the transcriptome equivalent to 55% of the total variability, with substantial effects not only in T cells, but also in NK cells and B cells (Fig. 1c, first principal component). That change in the gene-expression program can largely be attributed to changes in gene modules (Fig. 1d) representing cell-cell communication, response to stimulation, and signal transduction.

Despite the overall similarity of gene-expression patterns in different donor groups especially at steady-state, analysis of differentially expressed genes (DEG) revealed ~50 genes in the healthy donor vs. pSS and ~100 genes in the healthy donor vs. SLE, respectively, with significant expression differences (Fig. 1e, Supplementary Table 2). After T cell stimulation, DEG analysis was performed by first considering the difference in gene expression between T cell stimulation and unstimulated control, and in a second step comparing the increase or decrease of that expression difference between samples derived from healthy donors and pSS or SLE donors. Here, we detected highly significant DEG only in the healthy donor vs. SLE and only in T cells and B cells ( ~ 50 DEG in B cells and ~100 DEG in each T cell subset) (Fig. 1e, Supplementary Table 2). Hence, the analysis results were far more focused compared to steady-state conditions, again pointing to an increase in overall transcriptomic dissimilarity. Those DEG in T and B cells contained several important chemokines (CCL2, CCL3, CCL4, CXCL3), cytokines (LIF), and cytokine receptors (IL13RA1, IL27RA). Interestingly, IL27RA was detected as DEG in CTL after T cell stimulation, in line with recent findings on IL-27 signaling in T cells being associated with development of chronic inflammation in mice^25^. Remarkably, in both steady-state and T-cell stimulation conditions, DEG between healthy donors and patient-derived samples were cell-type exclusive, with very few exceptions.

Overall, the number of DEGs identified for pSS was much lower than for SLE. That points to pronounced molecular differences between SLE and pSS, which generally are considered to be both clinically and pathogenetically (type I interferon/B cell signatures) interrelated.

### Interferon-related genes show a strong SLE-specific response to T-cell stimulation in B cells

To further characterize the observed cell-type specific differences in gene-expression programs, we performed pathway over-representation analysis (Fig. 1f). For this purpose, instead of directly analyzing the DEG derived above, we implemented a rank-based analysis protocol^26,27^ specifically tailored to account for the skewed distributions that typically occur in sequencing count data lacking a direct negative control^28,29^, such as our steady-state data set. Here we applied in-sample centralization by median and standard deviation to the steady-state data, and for pathway analysis, we considered the top 50 up- and downregulated genes after ranking based on the false-discovery rate (FDR) for each condition (cf. Methods).

Quite remarkably, at steady-state, all cell types showed an increase in IFN-response gene activity for SLE patients, and CTL and B cells showed such an increase also in pSS patients (Fig. 1f). After T cell stimulation, a higher up-regulation of IFN-response genes compared to healthy donors was only significant for B cells in SLE patients (Fig. 1f). This observation suggests that T and NK cells show a pronounced response to CD3 stimulation also in healthy donors, but B cells retain a strong IFN response in the disease group compared to healthy controls. An overall increase in inflammatory genes as well as IFN-related genes upon stimulation was also confirmed by functional annotation of individual samples (Supplementary Fig. 2e). The most obvious differences in gene-expression programs after T cell stimulation, as compared to healthy donors, were Th cell associated down-regulation of several pathways associated with immune responses in SLE patients and an increase in activity of genes associated with cell cycle in samples of pSS patients (Fig. 1f). Further, B cells showed a highly significant up-regulation of a group of signaling pathway related genes exclusively in pSS patients (Fig. 1f).

Taken together, functional annotation reveals a range of cell-type and disease-specific traits, especially when comparing the response of samples from patients and healthy controls upon T cell stimulation.

### Clustering analysis reveals fine-tuned regulation of immune-related genes after T cell stimulation

**Fig. 2:**
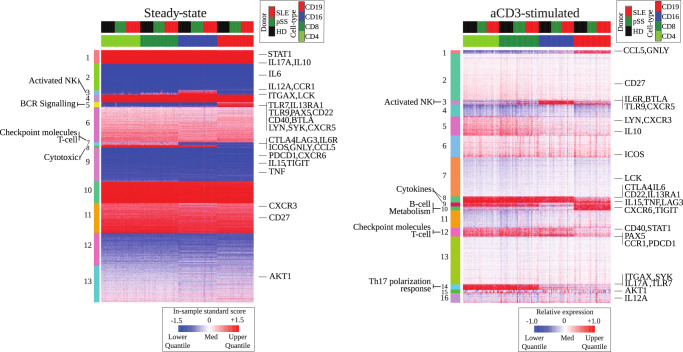
Clustering analysis reveals fine-tuned regulation of immune-related genes after T cell stimulation. Shown are gene-expression heatmaps sorted by gene similarity across indicated conditions. The left-hand side of each heatmap lists cluster numbers and annotations (see Supplementary Fig. 3 and Supplementary Table 1 for details), and the right-hand side labels highly relevant individual genes.

Unsupervised clustering analysis (Fig. 2) allowed us to gain a general overview over the two data sets, and confirmed the picture of an increase in sample-to-sample dissimilarity in the T cell stimulation protocol, where also a larger number of clusters was identified. We were able to identify functional annotations for most clusters (Supplementary Fig. 3), and the most obvious expression differences occurred for immune-cell related clusters between the sorted cell populations. For instance, cluster 3 was annotated as activated NK cells by the marker genes IL6R, CCR4, CCR6, BTLA, and indeed showed increased expression values in the NK cell population at both steady-state and upon T cell stimulation. Other clusters within the steady-state data indicated B cell receptor (BCR) signaling (cluster 5: BLK, BTLA, CD22, CD19, SYK, TLR), checkpoint molecules together with T cell activity (cluster 7: CD28, CD40LG, CTLA4, LTA, TRAF1) and cytotoxicity (cluster 8). After T cell stimulation, two clusters were detected with functional annotation towards cytokines (cluster 8: IL6, IL7, IL2, IL15, IFNG, and cluster 9: IL12, IL15, IRF6), with cluster 8 being active in all cell types except B cells and cluster 9 in all cells except NK cells. Interestingly, cluster 10 showed specific up-regulation in B cells, although it was annotated towards T-cell related gene sets, such as CD8+ TCR, T-cytotoxic, Th 1, Th17 differentiation (Fig. 2). Other important groups of genes, such as cluster 5, 6, 7, containing genes like IL10, ICOS, LCK, respectively, showed homogeneous up- or down-regulation after T cell stimulation in all analyzed cell types.

Overall, unsupervised clustering analysis confirmed that T cell stimulation via aCD3 not only resulted in a fine-tuned re-modulation of the immune-related transcriptional program across T cells, but also had a strong impact on NK cells and B cells.

### Interferon-related gene signatures of SLE and pSS patients depend on the activity of the autoimmune disease

The dysregulated activation of IFN and IFN-regulated pathways is well-known in SLE and pSS and represents a common target in clinical studies^30,31^. However, cell-type specific up-regulation of these pathways upon stimulation was not delineated so far. Here, we found that at steady-state, a strong IFN-driven gene signature was present across all cell types in SLE donors, but it was present only across NK cells and B cells in pSS patients. After T cell stimulation, that IFN-driven gene signature was only observed in B cells derived from SLE donors, indicating that only those cells showed a strong additional up-regulation of IFN-related genes compared to healthy donors (Fig. 1f).

**Fig. 3:**
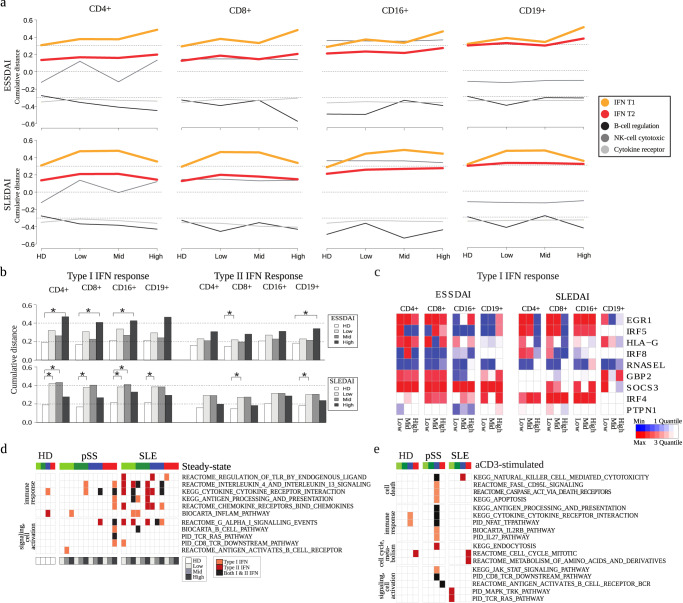
Interferon signatures show strong correlation with clinical activity among SLE and Sjögren’s patients, respectively. **a** Regulatory gene-expression dynamics alongside with clinical activity. Shown are regulatory levels of the indicated gene-sets in terms of the cumulative distance of the KS-test. Disease activity was classified based on the patient disease index scores ESSDAI for pSS and SLEDAI for SLE, respectively (see Table 1, Supplementary Fig. 4a and text for details). **b** Detailed analysis and statistical comparison of gene-expression dynamics for Interferon type I and II responses. **c** Driver genes of the type I interferon response dynamics. Genes were selected from the top 10% of sensitivity between activity levels. Type II interferon genes are shown in Supplementary Fig. 4c. **d**, **e** Co-expression analysis with respect to interferon-related genes for indicated conditions in steady-state **d** and after stimulation **e**, see Supplementary Table 1 for details. Co-expressed genes were calculated by permutation tests across all samples in the respective patient cohorts, and were subject to pathway-overrepresentation analyses. After stimulation, samples were analyzed without disease-activity resolution. For color code of cell type annotation, see the legend in Fig. 1b.

To explore the dynamics of IFN-related gene expression signatures in relation to the underlying disease activity in more detail, we used the disease activity indices EULAR Sjögren’s syndrome disease activity index (ESSDAI)^32^ and Systemic lupus erythematosus disease activity index (SLEDAI)^33^, respectively. Disease stages were classified as either “Low” (ESSDAI or SLEDAI of 1–3), “Moderate” (4–7) or “High” (8 and higher) (Supplementary Fig. 4a). In contrast to the CD3-stimulated condition (Supplementary Fig. 4b), we found that in steady-state conditions, both type I and type II IFN-response genes were strongly and significantly correlated with disease activity indices (Fig. 3a, b). In particular, type I IFN-response genes were among the top 5 pathways regarding association to disease activity (Supplementary Table 1). That strong disease-activity association of IFN-related genes was also present for instance in BCR genes, but not in other immune-related pathways including NK cell genes and cytokine receptor activity (Fig. 3a). Interestingly, the intensity of the IFN response consistently increased with disease activity among pSS patients, but had a maximum at “Low” and “Moderate” disease activity of SLE. Such a “bell-shaped response curve” has previously been noted for other systems, such as CD22 treatment with epratuzumab in SLE^34,35^. That pattern of either consistent increase with disease activity or of a maximum at “Low” and “Moderate” was also reflected in the level of individual IFN-related genes (Fig. 3c and Supplementary Fig. 4c), often in a cell-type specific manner.

Next, to further explore the implications of the association of IFN-related genes with disease activity, we sought to identify genes that show co-expression with IFN-related genes across all analyzed donors in the respective patient cohorts. For this purpose, we selected genes showing high similarity to IFN-related genes across all samples per cell type and disease condition, following an approach previously described for natural product screening^36^. Pathway over-representation analysis of the resulting gene set indicated that gene signatures revealing a change in metabolic and immunologic phenotype are strongly associated with the observed IFN response across pSS and SLE disease activity (Fig. 3d and Supplementary Fig. 4d). The strongest association with disease activity was observed for B cells in highly active pSS, where IFN alpha expression was associated with a range of the analyzed cell-signaling and immune-response pathways (Fig. 3d). That association was detected across all donors over a range of highly relevant pathway annotations, indicating that highly active pSS patients have an immune response that is strongly associated with upregulation of IFN alpha. After T-cell stimulation, we found strong association of IFN expression in particular with the NK cell response in pSS patients, across immune response and cell signaling, but also apoptosis related pathways (Fig. 3e).

Overall, analysis of IFN-related genes revealed pronounced cell-type and disease-specific traits already at steady-state condition. We detected a strong association of IFN-related genes with disease activity, which was connected to up-regulation of cell-signaling and immune-response pathways especially in B cells in pSS. Conversely, SLE patients showed a maximum in IFN-related genes at lupus activity considered Low or Moderate, and gene expression in B cells did not show strong correlation with IFN-related genes.

### Cell-cell communication analysis after T cell stimulation shows distinct interaction signals

**Fig. 4:**
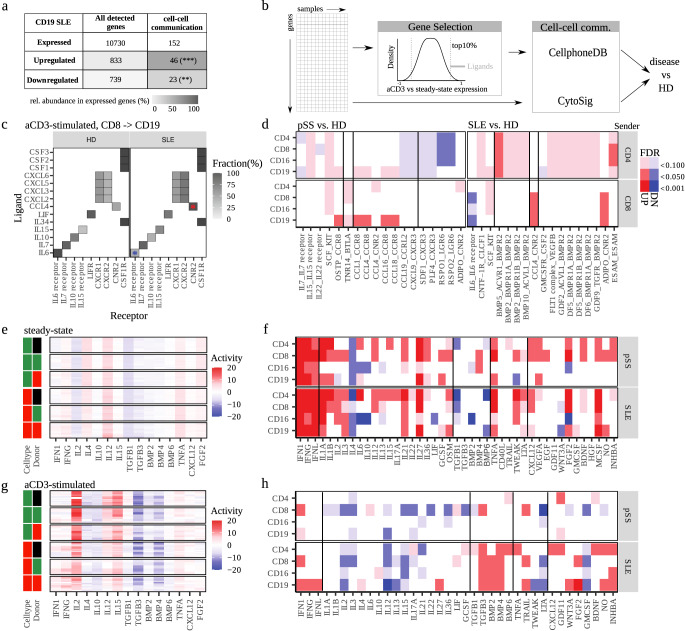
Cell-cell communication analysis reveals widespread re-modulation of cytokine and chemokine activity in SLE and primary Sjögren’s Syndrome. **a** Enrichment of cell-cell communication molecules in up- and downregulated genes after CD3 stimulation. Shown is the number and relative abundance (gray-scales) of detected genes as indicated, p-values indicate significant enrichment of cell-cell communication-related genes (Fisher’s exact test) among expressed genes (*p < 0.05, **p < 0.01, ***p < 0.001). **b** Workflow of cell-cell communication analysis. In CellPhoneDB analysis, we considered all interactions in the CellPhoneDB database for which ligand expression of a potential sender cells was in the top 10% of up-regulated genes after TCR stimulation, and receptors of a potential receiver cell were detected as expressed genes, for all donors and cell types. CytoSig analysis was carried out directly on the expression values of TCR stimulated relative to control samples (Fig. 1a), for all donors and cell types. **c**, **d** CellPhoneDB analysis. **c** Selection of ligand-receptor interactions between CTL and B cells in healthy donors and SLE **b**, shown is the fraction of donors where a potential interaction is detected for this condition. Asterisks correspond to up- and down-regulated ligand-receptor interaction between SLE and healthy donors fully depicted in **d**. **d** Comparison of healthy donors with SLE and pSS patients, respectively for selected cell-cell interaction pathways in the CellPhoneDB analysis. See Supplementary Fig. 5b for full data analysis. FDR-corrected *p*-values were calculated using Fisher’s exact test. **e**–**h** CytoSig analysis. **e**, **g** Predicted cytokine activity according to CytoSig for selected gene sets in CTL and B cells at steady-state and after stimulation, see Supplementary Fig. 5c, d for full analysis. For color code of cell type annotation, see legend in Fig. 1b. **f**, **h** Differences in cytokine activity between disease and control samples (steady state: Wilcoxon- test, aCD3-stimulated: T-test), see FDR legend in **d**.

Cell-cell communication pathways are known to have a pivotal role in autoimmune disease. Accordingly, we found evidence for differential regulation of cell-cell communication pathways in over-representation analysis of differential gene regulation (Fig. 1f) as well as disease-stage association (Fig. 3d). Moreover, we found that genes coding for cytokines and cytokine receptors were significantly enriched within the genes that were up- or down-regulated in disease compared to healthy donors after T cell stimulation, across all donors and cell types investigated (Fig. 4a and Supplementary Fig. 5a).

To explore disease-associated changes in cell-cell communication pathways more systematically, we employed two complementary approaches, the CellphoneDB database and the CytoSig package^37–39^ (Fig. 4b). CellphoneDB is a curated list of known cell-cell communication pathways, which we employed to identify potentially active signaling pathways by co-regulation in the analyzed patient cohorts after T cell stimulation. For instance, considering Th cells as sender cells and B cells as receiver cells, we identified the CCL4 to CNR2 receptor pathway in 5 out of 13 healthy donors and 8 out of 9 SLE donors, which is a significant increase in occurrence (Fig. 4c). Overall, we identified 33 up-regulated and 2 down-regulated cell-cell communication pathways comparing healthy donor to SLE patient samples, and 10 up-regulated and 14 down-regulated pathways in pSS patients (Fig. 4d and Supplementary Fig. 5b). Notably, activation-induced IL-6 signaling was down-regulated in SLE patients especially in CTL and B cells, in line with a previous report on TLR9 activation of SLE B cells^40^. Further, pSS patients showed a broad increase in IL-15 and SCF_KIT signaling (Fig. 4d). Quite interestingly, SLE patients up-regulated the rather anti-inflammatory BMP pathways, while pSS donors showed widespread remodeling of chemokine signaling (Fig. 4d, Supplementary Fig. 5b).

The CytoSig tool quantifies the increase or decrease in expression of cytokine-induced genes in potential target cells of cell-cell communication (Fig. 4b). Apart from identification of target cells, this method also serves to detect cytokine signaling activity with higher sensitivity compared to direct analysis of cytokine transcripts in sender cells, since cytokine transcript levels are typically low and expression of cytokine genes does often not fully reflect cytokine release activity. Not surprisingly, the CytoSig analysis revealed moderate activity of many cytokine signals at steady-state (Fig. 4e and Supplementary Fig. 5c). Nevertheless, we found significant differences between pSS or SLE patients and healthy donors for a range of inflammatory cytokines including interferons and TNF-alpha (Fig. 4f). Accordingly, the type II cytokine IL-4 and cytokines of the anti-inflammatory TGF-beta and BMP family showed decreased activity in disease, while the pleiotropic cytokine IL-6 showed increased activity in Th cells and decreased activity in NK cells, for both pSS and SLE patients as compared to healthy donors.

As expected, upon T cell stimulation we found strong increase in the activity of gene signatures related to inflammatory cytokines, including IFNs, IL-2, and IL-15, while we found down-regulation of genes related to anti-inflammatory cytokines, such as TGF-beta (Fig. 4g and Supplementary Fig. 5d). Similar to the CellphoneDB approach, we next analyzed differences in the response to T cell stimulation between healthy donors and patients by comparing the cytokine activity values across samples from all donors (Fig. 4h). Quite interestingly, in contrast to IFN-related genes, where the expression signature already found in our more general over-representation analysis was confirmed (Fig. 1f), many other cytokine-related gene signatures were down-regulated in disease (Fig. 4h). In line with that, several anti-inflammatory TGF-beta related pathways showed up-regulation especially in SLE donors.

**Fig. 5:**
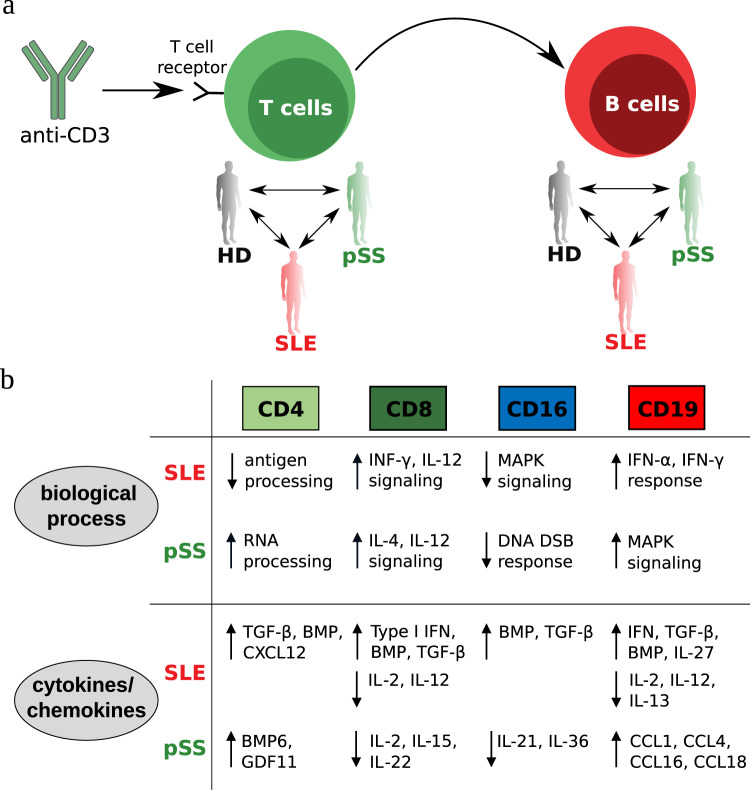
Summary of cell type-specific modulations in pSS and SLE patients after T cell stimulation. **a** Graphical synopsis of the experimental design. After T-cell stimulation via aCD3, disease-specific characteristics became apparent, especially within cell-cell communication patterns between healthy donors (HD), pSS, and SLE patients. **b** An overview of altered, cell type-specific processes after aCD3 stimulation. (top) biological processes, (bottom) and differentially regulated cyto- and chemokines.

Overall, we found that T cell stimulation has profound effects on the transcriptome not only in T cells, but also in NK cells and B cell (Fig. 5a) (Figs. 1b, c and 2). Moreover, our systematic analysis of cell-cell communication pathways revealed substantial re-modulation of gene regulation and cell-cell communication within the two autoimmune diseases in a cell-type and disease-specific manner (Fig. 5b). While up-regulation of IFN-related genes primarily in B cells of SLE patients was confirmed, we also detected down-regulation of a range of important pro-inflammatory cytokine pathways and up-regulation of anti-inflammatory pathways in disease.

## Discussion

This study has undertaken a functional transcriptional analysis of isolated immune-cell subsets in SLE and pSS compared to controls, both at steady-state and upon T-cell stimulation via aCD3, which served as a surrogate of a T cell specific immune activation. In this study, we focused on B cells, NK cells, and CD4+ and CD8+ T cells, although changes in abundance and gene-expression signatures in SLE and pSS patients have also been reported for other populations such as plasma cells and monocytes^15^.

At steady-state, all 4 analyzed lymphocyte subsets from SLE patients displayed a pronounced IFN-response gene activity, which was also found in CTL and B cells in pSS, in line with previous bulk PBMC analyses^13^. The high degree of transcriptional similarity between donor groups in all four cell types exacerbated direct conclusions regarding other functional transcriptomic differences in disease, yet further analysis of IFN-response genes revealed a series of partially unexpected results. First, by detailed assessment of expression similarity with IFN-response genes, we detected a range of significant functional differences in disease, most notably regarding cell activation and immune response for B cells in pSS at high disease activity. Second, by tracking the cumulative distance across samples, we found a strong and significant correlation of IFN-related genes with disease activity. For pSS patients, a positive correlation with disease activity as assessed by the ESSDAI was detected, whereas in SLE patients, IFN signatures were increased at low and moderate disease activity but dropped again at high disease activity, for all cell types except NK cells. That finding is somewhat paradoxical in the light of the otherwise stronger IFN-association observed for SLE compared to pSS patients, and can potentially be attributed to exhaustion or chronification mechanisms occurring in high-stage SLE immune cells^41,42^. Interaction between type I IFN and B cell parameters in SLE patients were previously observed in response to Jak blockade (baricitinib), where decrease of anti-dsDNA antibodies was associated with reduced IFN gene signatures^43^. Moreover, lymphocytes from SLE patients were described to carry the most pronounced status of anergy compared to pSS as well as rheumatoid arthritis patients^42^, which may further contribute to the observed differences between SLE and pSS.

Upon T cell stimulation, both healthy donors and pSS as well as SLE patients up-regulated IFN-dependent genes. Intriguingly, activated T cells provided signals for B cells without the presence of any exogenous antigenic stimuli and co-stimulation. That finding is in line with previous work suggesting antigen-independent activation of B cells by activated T cells as a potential mechanism for the development and maintenance of autoreactive germinal centers and extrafollicular activation, ultimately leading to and maintaining chronic autoimmune diseases^44^.

Importantly, we found that the T cell stimulation protocol emphasized transcriptomic differences between both patient groups and healthy controls, in many cases unmasking expression signatures that could not be detected in the steady-state condition. In the interpretation of these results, it is important to consider the methodological differences: in contrast to the steady-state situation, the stimulation protocol allowed us to assess gene expression in terms of relative expression values compared to PBS controls from the same donors. That work-flow contributed to avoiding the common problem of skewed distributions of expression values in directly processed transcriptomic sequencing data^28,29^, and therefore lead to an increase in statistical power in subsequent analysis steps. Hence, the observed increase in dissimilarity between samples after applying the T cell stimulation protocol goes along with a higher sensitivity for detecting such differences. Here, the better resolution obtained from the T cell stimulation protocol became apparent already by PCA on the global transcriptomic scale, and gave rise to ~100 highly significant DEG for Th cells and CTL and ~50 DEG for B cells. Further, T cell stimulation revealed a number of functional transcriptomic differences, in particular increased DNA stress in Th cells and decreased signaling activity in B cells in pSS, and decreases immune response activity in Th cells in SLE.

Our analysis of cell-cell communication genes further emphasized the finding that cells from SLE and pSS patients have many common properties at baseline, whereas T cell stimulation revealed disease-specific differences. CytoSig analysis at steady-state revealed strong up-regulation of signaling through IFNs and other inflammatory cytokines, as well as down-regulation of IL-4 and anti-inflammatory TGF-beta and BMP pathways, across both SLE and pSS for most of the analyzed conditions. CellphoneDB analysis after T cell stimulation revealed for instance CCL4 to CNR2 receptor pathway interaction exclusively in SLE. This pathway was found in 8 out of 9 SLE donors, and has so far not been delineated in either disease. Further, anti-inflammatory BMP pathways were elevated primarily in SLE, consistent with earlier studies^45^, and chemokine signals such as CXCL9-CXCR3 were down-regulated primarily in pSS. CytoSig analysis after T cell stimulation confirmed that picture, and in addition revealed selective down-regulation of a number of cytokines including IL-12, IL-22, IL-36 in only one of the diseases and primarily for specific cell types. Hence, a distinct pattern of up- and downregulated pathways for SLE and pSS patients further substantiated the notion of distinct pathogenic drivers. Further, our data is consistent with previous reports indicating reduced cytokine levels in SLE^40^, which may represent a part of the immune abnormalities of the entities studied here.

Single-cell sequencing technologies have the benefit of unbiased cell-type classification and therefore offer the opportunity to discover disease-specific cell types or states, but that comes at the cost of substantially lower sequencing depth compared to bulk transcriptomic sequencing. Indeed, previously conducted single-cell studies of pSS and SLE patients have revealed high levels of heterogeneity as well as differences in the abundance of immune cells between patient groups, but could detect only a limited range of expression differences. Moreover, it proved challenging to unambiguously separate lymphocyte subpopulations especially for T cells^15,16^. Transcriptomic analysis of sorted immune-cell populations is a complementary approach with a high potential for discovery of transcriptomic differences within specific cell types^21,22,46^, yet at the cost of limiting the analysis to a set of predefined cell types. Our analysis suggests that in addition to deep sequencing using an appropriate sorting strategy, a perturbation protocol such as the here applied T cell stimulation is instrumental to gain molecular insight into heterogeneous diseases such as SLE and pSS.

Taken together, our data highlight individual variations of transcriptional activity between cell types as well as diseases. At steady-state, the different cell types are characterized by autoimmune response, signaling, and activation, possibly representing the underlying disease-related tonicity. After T cell stimulation, these regulatory signatures revealed footprints for early cell-type differentiation and an autoimmune-disease related response pattern, suggesting subtle differences in the T helper cell response between SLE and pSS. Therefore, the gene-expression context and its functional impact underlying SLE and pSS needs to be delineated in detail in future research, as an important step towards improved understanding of the pathogenic function of individual immune cells, and to improve clinical applications.

## Methods

All methods were carried out in accordance with relevant guidelines and regulations.

### Study participants

Lithium Heparin (LiHep)-anticoagulated blood was withdrawn from a total of 24 healthy donors, 25 SLE patients and 23 pSS patients. All patients fulfilled the disease classification criteria^23,24^. We assessed the disease activity using SLE disease activity 2000 [SLEDAI-2K] for SLE^33^ and EULAR Sjögren’s syndrome disease activity index [ESSDAI] for pSS^32^. Low disease activity was applied to ESSDAI or SLEDAI of 1–3 following the definition of low lupus disease activity score^47^. High disease activity was defined as ESSDAI or SLEDAI > 8, as often used to classify active patients in clinical studies. Patients with ESSDAI or SLEDAI 4–8 were considered moderate active. The study was approved by the local ethics committee of Charité Universitätsmedizin Berlin in accordance with the declaration of Helsinki and written informed consent was obtained from all patients and controls. Information on patient demographics and medication as well as inclusion criteria are collected in Table 1.

### Cell culture

PBMCs were isolated from LiHep-anticoagulated blood: blood from two 9 ml LiHep tubes were combined and PBS (Thermo Fisher Scientific) was added up to a volume of 35 ml. This mixture was layered over 15 ml Ficoll Paque Plus (GE Healthcare) and subjected to density gradient centrifugation (20 min at 600 × g, room temperature, without brake)^41^. The collected mononuclear cells were washed twice with PBS and counted for further experiments. After isolation of PBMCs, cells were rested for 30 min at 37 °C in ImmunoCult-XF T cell expansion medium (Stemcell). Wells of a 96-well plate were coated with 0.1 µg/ml anti-CD3 (OKT3, eBioscience) for 2 h at 37 °C or PBS as control, washed two times with PBS, blocked with PBS/1% BSA for 30 min and washed two times with PBS. 1 × 106 PBMCs were seeded per well and incubated at 37 °C and 5% CO_2_ in a humidified incubator for 18 h. Cell viability after stimulation was above 95%.

### Fluorescence-activated cell sorting

For baseline sorting, 3–5 × 10^6^ PBMCs were used. For cell sorting after 18 h incubation, cells from wells with same conditions were pooled and stained. The following anti-human antibodies (clone, manufacturer) have been used: Brilliant Blue (BB)515-conjugated anti-CD3 (UCHT1, BD Biosciences), Phycoerithrin (PE)/Dazzle594-conjugated anti-CD4 (SK3, Biolegend), Brilliant Violet (BV) 510-conjugated anti-CD7 (M-T701, BD Biosciences), Peridinin-chlorophyll-protein complex (PerCp)-conjugated anti-CD8 (SK1, Biolegend), BV421-conjugated anti-CD14 (M5E2, Biolegend), Allophycocyanin-Fire750 (APC/Fire750)-conjugated anti-CD16 (3G8, Biolegend), PE-Cyanine 7 (PE-Cy7)-conjugated anti-CD19 (SJ25C1, BD Biosciences), APC-conjugated anti-CD25 (2A3, BD Biosciences), PE-conjugated anti-CD127 (HIL-7R-M21, BD Biosciences). 4,6-Diamidine-2-Phenylindole (DAPI) (Molecular Probes) was added to stained cells to identify dead cells prior to acquisition. Cells were subsequently sorted with a BD FACS Aria I or II (BD Biosciences). A detailed gating strategy for sorting can be found in Supplementary Fig. 1. Briefly, lymphocytes and single cells were identified by their scatter properties. For baseline sorting, CD127+ CD25− conventional T cells of both CD4+ and CD8+ T cells were sorted in addition to CD19+ B cells and CD16+ CD7+ NK cells. After 18 h, total CD4^+^ and CD8^+^ T cells, CD19+ B cells and CD16+ CD7+ NK cells were collected. Cells were sorted into Arcturus PicoPure Extraction Buffer (Thermo Fisher Scientific) and stored at −80 °C until processing for transcriptome analyses.

### RNA sequencing

The PicoPure RNA Kit (Thermo Fisher) was used for the purification of total RNA from cells according to the manufacturer’s protocol. Library generation has been completed using the SMARTer cDNA Synthesis and Nextera XT chemistry. With 500 pg total RNA, cDNA pools were prepared using the SMART-Seq v4 Kit (Clontech Laboratories) to generate full-length cDNA from total RNA. The resulting cDNA was then amplified via long distance PCR of 10 cycles. Sequencing libraries are then created using the Nextera XT method (Illumina) with 150 pg of cDNA as input. Cluster generation and sequencing of libraries will utilize Illumina HiSeq V4 chemistry and instrumentation.

### Data processing procedures

The BCL (Base calling) files were converted to FASTQ (raw sequence reads) files using the bcl2fastq v2.17.1.14 tool provided by Illumina (https://support.illumina.com/downloads.html). In order to estimate gene-expression levels, paired-end RNA-sequence reads were mapped to the reference of the Homo sapiens genome assembly GRCh38 (hg38) with the STAR aligner version 2.5.2a using default mapping parameters^48^. Aligned reads were subsequently quality checked with FastQC and MultiQC version 1.7^49^ tools. Then the numbers of mapped reads for all Reference sequence transcript variants of the genome were combined into a single value by the SAMTOOLS package^50^. Raw read counts were normalized to the FPKM (Fragments Per Kilobase Million) standard. Within each batch, bad quality samples were excluded based on three measures. While the number of uniquely mapped reads was not allowed to be below 50%, weak or wrong cell type signature enrichments inferred by the BioQC package^51^ were excluded. Lastly, outliers detected by principal component analysis (PCA) were removed from downstream analysis. Solely PCA outliers were kept, if no evidence for a technical issue was found. Further, we restricted the analysis to protein-coding genes with total read-counts across samples within the 45–99.5 percentile range, in order to include 19 candidate genomic markers. The expression profile of aCD3-stimulation was calculated as relative gene expression, that is FPKM values of stimulated samples were normalized by FPKM values of the PBS control per each sample independently. Steady-state samples were processed using a slightly different method, since the steady-state samples were not derived from negative control samples, and therefore the resulting gene-expression distributions are subject to skewness^28,29^. Thus, we applied in-sample centralization by median and standard deviation from normalized FPKM counts for reducing bias effects. Overall, gene-expression profiles consist of 10,835 genes across samples both from the steady-state and the T cell stimulation protocol.

### Pathway annotation

Differentially expressed genes in the steady-state data and after aCD3 stimulation were identified using standard statistical procedures (see section Statistics below). A pathway over-representation analysis of the C2 MSigDB entries was performed on the top 50 as well as the bottom 50 genes according to false-discovery rate, rather than directly on the DEGs. In the first step, the gene-sets were reduced to the genes expressed in our data set. To measure the significance of a pathway annotation, we applied the hypergeometric test, and we manually set the *p*-value to 1 if the input gene set contained less than three or more than 300 entries, to focus on biologically meaningful results and thereby enhance statistical power.

### Co-functional analysis

Following the well-known gene-set enrichment analysis (GSEA) protocol^52^, we considered the C2 MSigDB gene-sets reduced to genes expressed in our data, and removed gene-sets containing fewer than three and more than 500 entries. Again following the GSEA work-flow, similarity was then quantified using the cumulative distance with respect to gene-sets, that is the maximal difference between the cumulative distribution functions of the gene-set of interest and of the relevant control group. That measure is also often used in the context of the Kolmogorov-Smirnoff (KS) test. To systematically analyze genes showing a co-functional behavior towards IFN responses as well as immune related gene-sets, we applied guilt-by-association (GBA)^53,54^ to the similarity between gene-sets. The GBA approach is common for dissecting genomic associations such as protein interaction and predicting unknown functions by expression profile. Here, we first computed the Euclidean distance of gene-expression values of selected IFN response gene-sets (MSigDB C2) to all other genes for all samples included. Second, to define a group of genes expressing similar IFN responses, a random permutation test (*n* = 10,000, FDR adjusted) was performed to detect genes that show a significantly lower distance to a particular IFN response gene, for a particular experimental condition (e.g., CD4+ Th cells in SLE) as compared to the full set of genes across all conditions. Finally, the union of detected genes along the selected set of IFN response genes, for each condition, was taken as input for pathway over-representation analysis (see above).

### Cluster annotation

We aimed to identify gene-modules by using a k-means clustering approach. After unsupervised clustering, we heuristically selected clusters containing less than 500 genes to ensure the statistical viability of the pathway annotation. Based on the expression patterns of clinically relevant markers, we manually annotated the gene clusters. The used marker genes are annotated in Supplementary Fig. 3. See Supplementary Table 1 for detailed results of unsupervised clustering.

### Cell-cell communication analysis

In order to dissect communication patterns between different cell types after aCD3 stimulation, two different approaches were used. For the first approach, we used the curated list of ligand-receptor interactions (Interaction Input) collected in the CellPhoneDB database^38^. After sorting the columns for ligands and receptors, respectively, we extracted CC-signals per donor. Here solely donor samples were considered where all sorted bulk-data sets passed the QC step. Generally, all cell types were allowed as receivers, whereby only T cells (CD4 and CD8) were included as sender cells. Using the CellPhoneDB database, we focused on the ligands which were expressed within the top 10% of the genes based on the distribution of the steady-state normalized transcription data. On the receptor site, only expressed genes were included. After collecting all the possible interaction signals across all sender-receiver pairs, statistically significant differences between disease (SLE and pSS) and healthy donors were calculated using a Fischer’s Exact test per detected ligand-receptor pair. To allow for a functional assignment of the different CC-signals, we grouped the results based on the following order: Cytokines, TGF and related entries, TNF and related entries, chemokines, growth factor, and others. In a second approach we employed the CytoSig tool^39^ to predict cytokine target activity after aCD3 stimulation. Using the steady-state normalized expression data as input, we extracted the Z-score activity from the CytoSig online tool. In order to also state significant differences between disease states and healthy donors across target cell types, t-test statistics per cytokine were calculated. Due to the distribution of Z-score values, a Wilcoxon test was calculated for the steady state data. Similar to the previous approach, the entries were ordered according to: interferons, cytokines, TGF and related entries, TNF and related entries, chemokines, growth factor, and others.

### Software and biotypes

R (Version 4.1.3) was used for all statistical analyses, mainly relying on packages such as: Tidyverse, Plyr, Pheatmap, RColorBrewer, VennDiagram, org.Hs.eg.db, EnsDb.Hsapiens.v86, iGraph, Bioconductor, ggplot2. All gene symbols were updated by the org.Hs.eg.db Version 3.14.0 and the Biotypes were collected from the EnsDb.Hsapiens.v86 Version 2.99.0. Key matching variable is the ENTREZID.

### Statistical analysis

Functional enrichments within gene sets were calculated using a hypergeometric test, whereby the regulatory score was calculated using a one-side Kolmogorov–Smirnov. Differentially expressed genes as well as different cytokine activities were inferred using a Wilcoxon test. The obtained *p*-values were adjusted for multiple testing using the Benjamini-Hochberg method. If not indicated otherwise, all analyses were conducted at the 10% FDR level.

### Reporting summary

Further information on research design is available in the Nature Research Reporting Summary linked to this article.

### Supplementary information

Supplemental Material

Supplementary Table 1

Supplementary Table 2

Reporting Summary

## Data Availability

RNA-sequence and processed matrix data have been deposited in the ArrayExpress database at EMBL-EBI (www.ebi.ac.uk/arrayexpress) under accession number E-MTAB-12203.

## Code availability

All scripts used to generate the paper results and Figures are provided on github (https://github.com/Kwon-DRFZ/Lupus).
