# Supplementary material for: Transcriptional profiling upon T cell stimulation reveals down-regulation of inflammatory pathways in T and B cells in SLE versus Sjögren’s syndrome
Source: NPJ Syst Biol Appl. 2023 Dec 15;9:62. doi: 10.1038/s41540-023-00319-z (PMC10724199; doi:10.1038/s41540-023-00319-z)
Supplement: Supplementary file 1 — Supplemental Material [file 41540_2023_319_MOESM1_ESM.pdf]

Supplementary Materials for

Transcriptional profiling upon T cell stimulation reveals down-regulation of inflammatory pathways in T and B cells in SLE versus Sjögren's syndrome

Gino Kwon, Annika Wiedemann, Lisa M. Steinheuer, Ana-Luisa Stefanski, Franziska Szelinski, Tomas Racek, Andreas Philipp Frei, Klas Hatje, Tony Kam-Thong, David Schubert, Thomas Schindler, Thomas Dörner*, Kevin Thurley*

*Corresponding authors. Email: thomas.doerner@charite.de, kevin.thurley@uni-bonn.de

The PDF file includes:

Supplementary Figures 1- 5

Legends for Supplementary Tables 1 and 2

Other Supplementary Material for this manuscript includes the following:

Supplementary Tables 1 and 2

Supplementary Figures

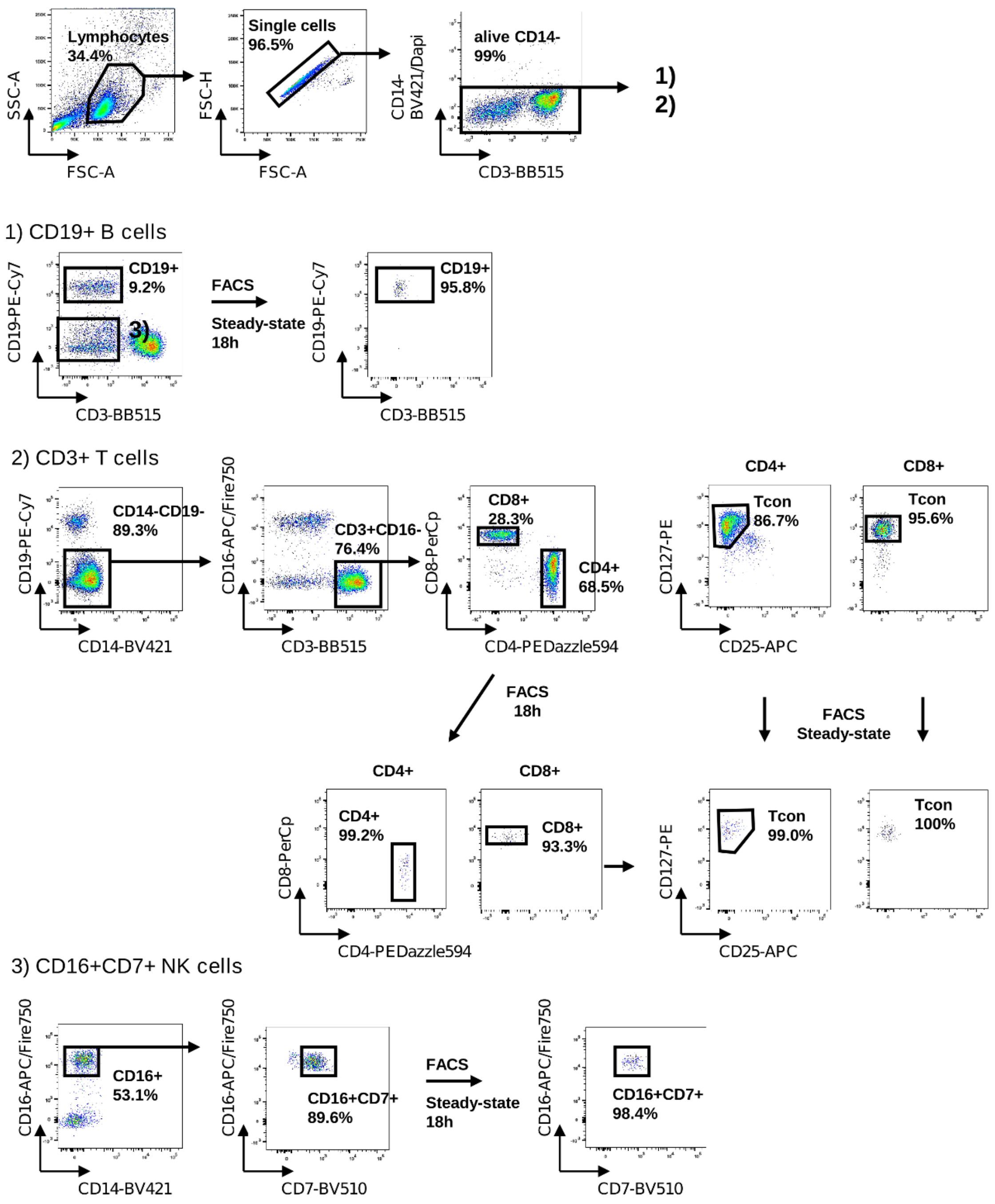

Supplementary Figure 1: Sorting strategy applied before transcriptomic analyses. Lymphocytes and single cells were identified based on their scatter properties and dead cells as well as CD14+ monocytes were excluded. B cells were gated by CD19 expression, NK cells by lack of CD3 and CD19 and positive staining for CD16 and CD7. T cells were identified by the lack of CD14, CD16, CD19 and positive expression of CD3 and were further subdivided into CD4+ and CD8+ cells. At baseline, conventional CD127+CD25- cells (Tcon) in both T cell subsets were sorted.

a

	Steady-state			aCD3-stimulated			
	HD	pSS	SLE		HD	pSS	SLE
CD4+	24	20	25	CD4+	15	10	13
CD8+	23	20	24	CD8+	15	13	14
CD16+	22	22	24	CD16+	13	11	13
CD19+	22	20	23	CD19+	14	10	15

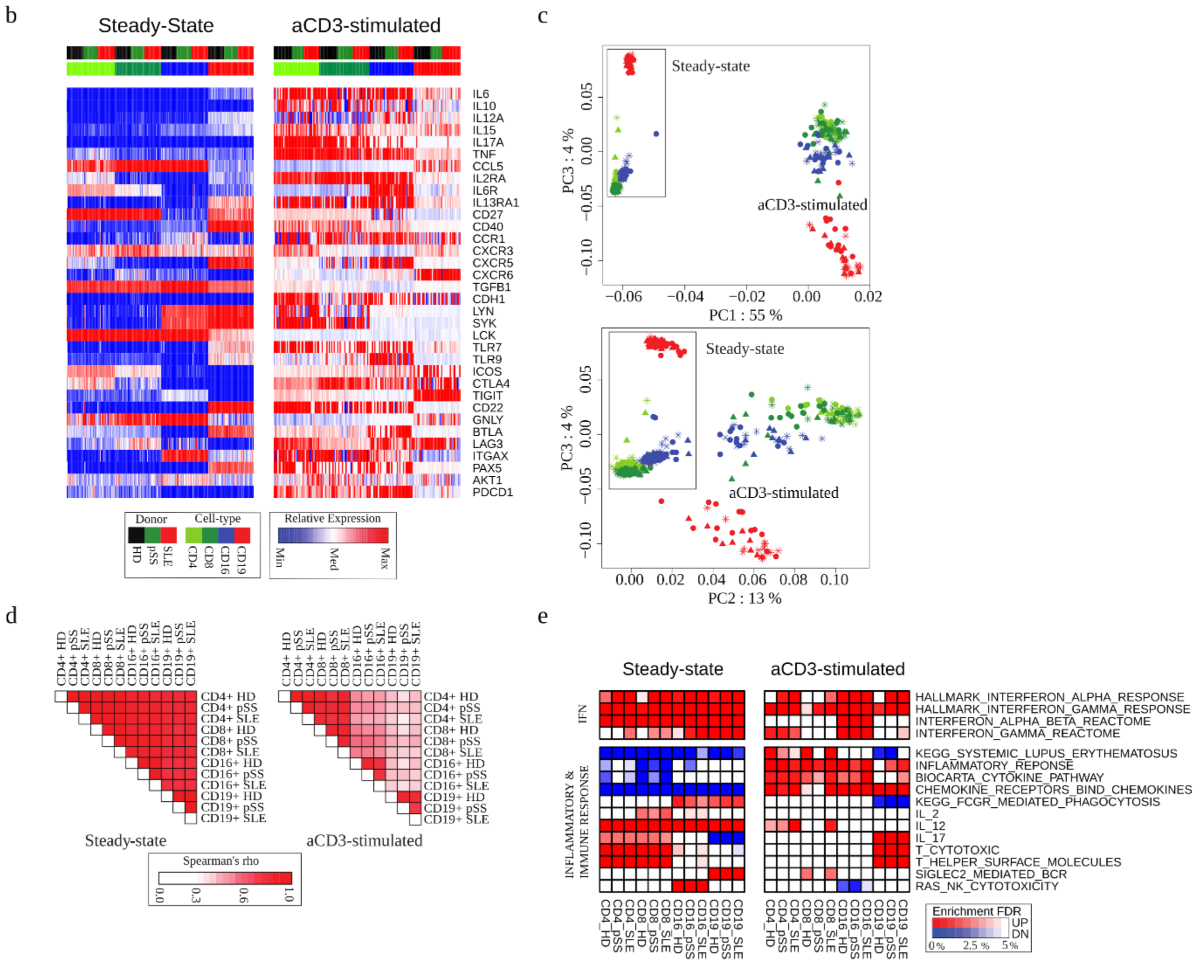

Supplementary Figure 2: Extended data to Figure 1.
(**a**) Overview of samples per condition used for RNA sequencing, see Supplementary Table 1 Tab 1 for details. Note that all aCD3-stimulated samples are supplemented by corresponding PBS controls, giving rise to 581 sequencing samples in total. (**b**) Expression of prior genes in donor-resolution (expanded Figure 1b). (**c**) Extended principal component analysis (see Figure 1c) showing PC3 vs. PC1 and PC2, respectively. (**d**) Computed Spearman's rank correlation coefficient from the average expression ranking of each condition, see Supplementary Table 1 Tab 4 for details. (**e**) Functional annotation of individual samples.

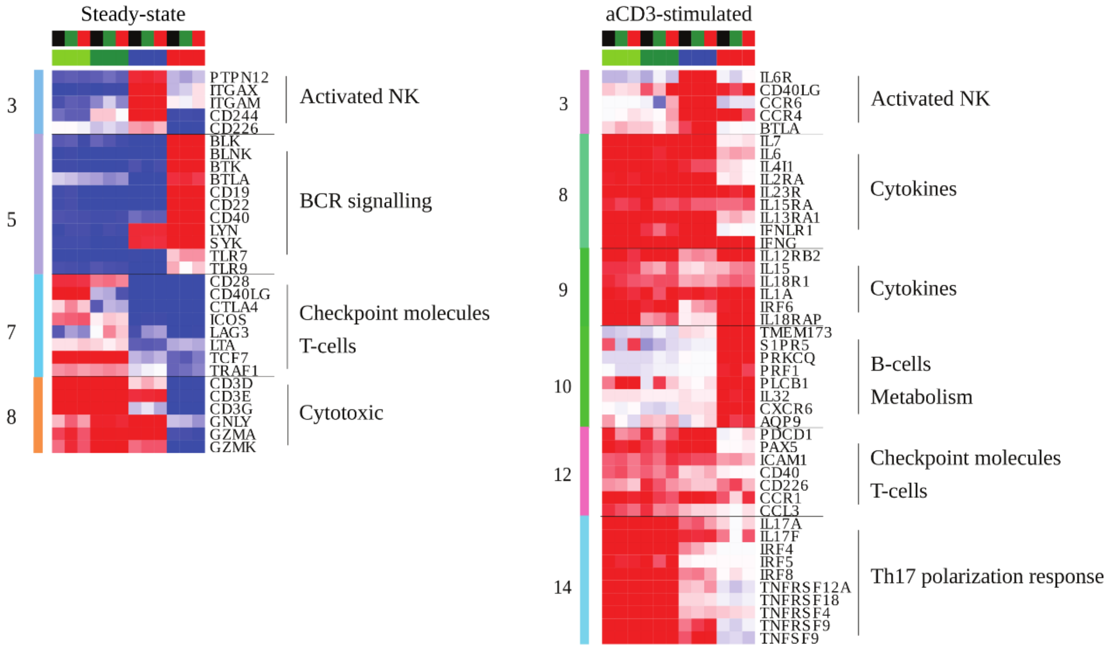

Supplementary Figure 3: Extended data to Figure 2.
Shown are expression values of marker genes for the cluster annotation adopted in Figure 2 in indicated conditions for the steady-state and aCD3-stimulated samples. See Figure 1b for color code of cell type and donor annotation and for expression levels, and see Supplementary Table 1, Tab 5 and 6, for cluster membership and detailed pathway annotation.

a

Number of sample per Index-stage
Steady-state

		CD4+	CD8+	CD16+	CD19+
HD		24	23	22	22
ESSDAI	Low	5	6	6	5
	Mid	7	5	6	5
	High	3	3	3	3
SLEDAI	Low	13	12	12	12
	Mid	6	6	6	5
	High	4	4	4	4

b

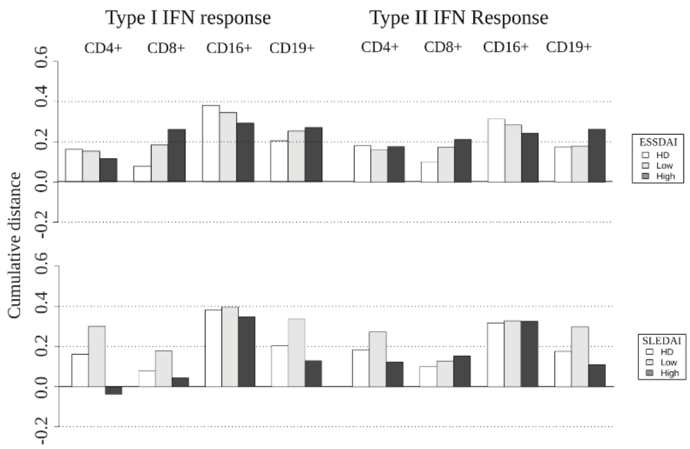

c

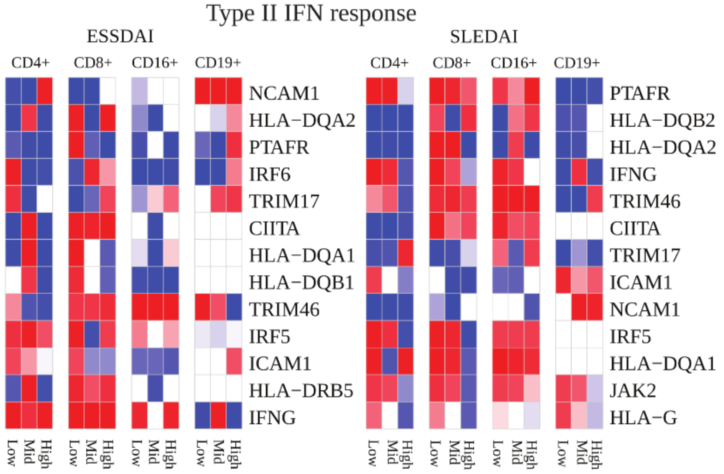

d

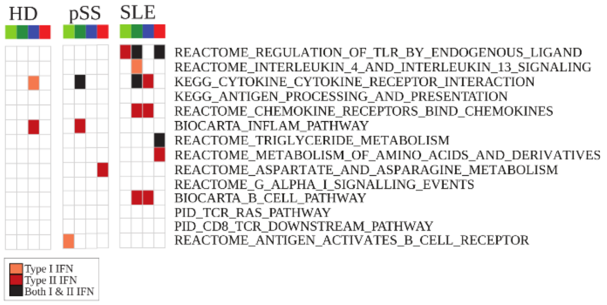

Supplementary Figure 4: Extended data to Figure 3.
(**a**) Numbers of samples in the steady-state condition with annotated grouping according to disease score for SLE and pSS patients. (**b**) Analysis of gene-expression dynamics for IFN type I and II responses after aCD3 stimulation, analogous to Figure 3b. (**c**) Driver genes of the type II IFN response genes were selected under the top 10% of sensitivity between stages, analogous to Figure 3c. (**d**) Analysis of gene-similarity with IFN at steady-state without disease-stage resolution, analogous to Figure 3d and e.

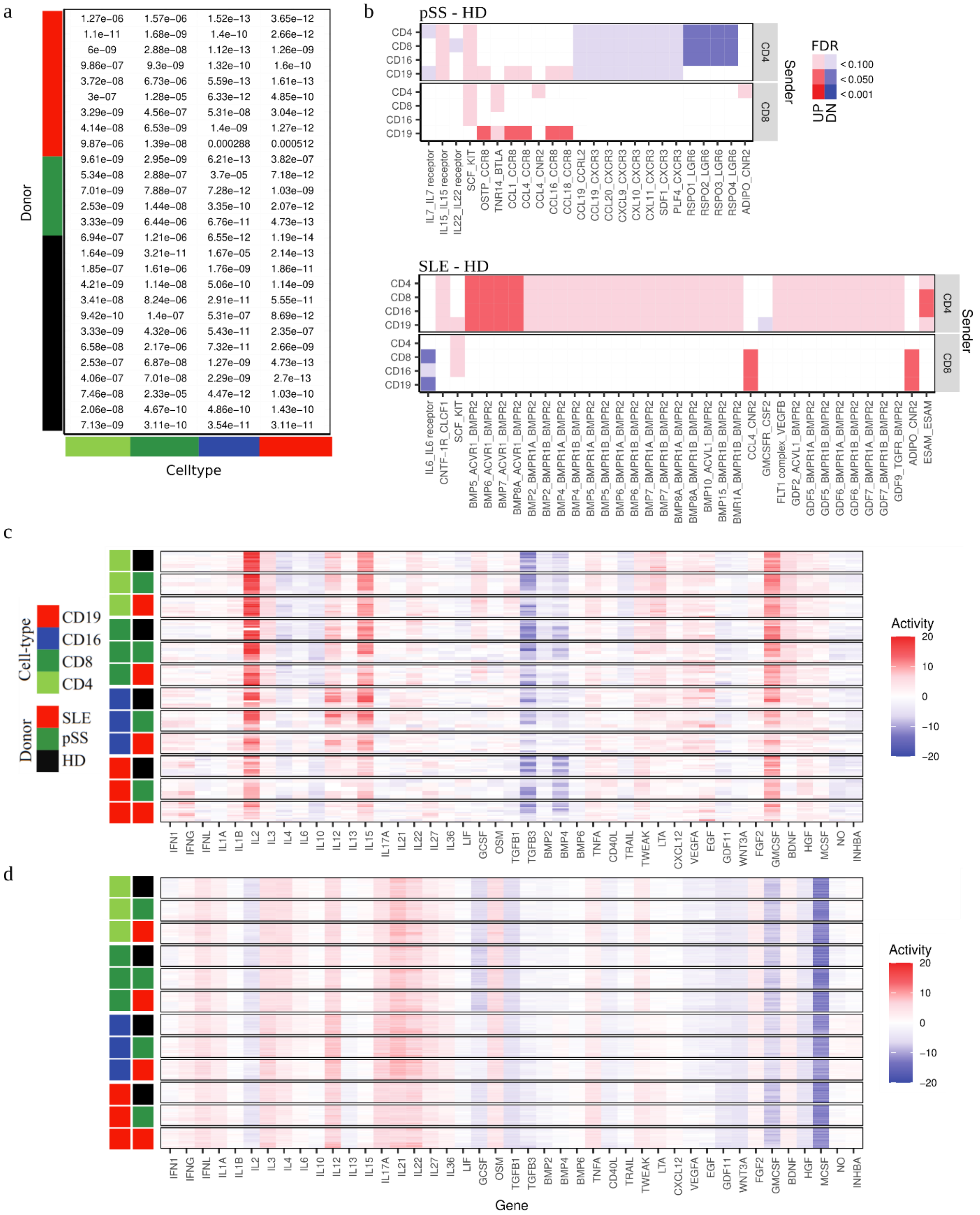

Supplementary Figure 5: Extended data to Figure 4.
(**a**) Significant enrichment of cell-cell communication molecules in up- and downregulated genes across all donors and cell types (Fischer's exact test). (**b**) Statistical analysis of ligand-receptor interactions for CTL and Th cells as sender, respectively (expanded Figure 4d). The entries were grouped according to cytokines, TGF-related terms, TNF-related terms, chemokines and other growth factors. (**c-d**) Predicted cytokine activity according to CytoSig across all cytokines and donors at steady-state (c) and after aCD3-stimulation (d) (expanded Figure 4e and g).

Supplementary Table Legends

Supplementary Table 1 (separate file): Supplementary data accompanying the transcriptomic analysis.
(**Tab 1**) Overview of all sequencing samples including technical and experimental specifications. (**Tab 2**) Additional information of the PCA-analysis such as the eigenvectors (A) and component scores (B) (related to Figure 1c and Supplementary Figure 2c). (**Tab 3**) Functional annotation of the highly variable genes per PC component, i.e. top 50 and bottom 50 genes (Figure 1d). (**Tab 4**) Sample similarity using the spearman correlation coefficient (Supplementary Figure 2d). (**Tab 5**) Cluster membership per gene at steady-state and after aCD3 stimulation. (**Tab 6**) Functional annotation of the gene clusters (Supplementary Figure 3b). (**Tab 7**) Top 50 up- and down-regulated genes for indicated conditions. (**Tab 8**) Functional annotation of the top up- and down-regulated genes (Figure 1f). (**Tab 9**) Calculated IFN-similarity indices at steady-state (with disease-stage index) and after aCD3-stimulation, the entries were filtered using a p-value cutoff of 5%. (**Tab10**) Functional annotation of IFN-similarity genes (Figure 3d and e).

Supplementary Table 2 (separate file): An overview of the differentially expressed genes.
All DEGs detected in the steady state data and after aCD3 stimulation are listed, including FDR as well as the fold change. The tabs indicate the DEGs per cell type.
