# Supplementary material for: Transcriptional profiling upon T cell stimulation reveals down-regulation of inflammatory pathways in T and B cells in SLE versus Sjögren’s syndrome
Source: NPJ Syst Biol Appl. 2023 Dec 15;9:62. doi: 10.1038/s41540-023-00319-z (PMC10724199; doi:10.1038/s41540-023-00319-z)
Supplement: Supplementary file 4 — Reporting Summary [file 41540_2023_319_MOESM4_ESM.pdf]

Corresponding author(s): Thomas Dörner, Kevin Thurley

Last updated by author(s): Sep 11, 2023

Reporting Summary

Nature Portfolio wishes to improve the reproducibility of the work that we publish. This form provides structure for consistency and transparency in reporting. For further information on Nature Portfolio policies, see our Editorial Policies and the Editorial Policy Checklist.

Statistics

For all statistical analyses, confirm that the following items are present in the figure legend, table legend, main text, or Methods section.

n/a	Confirmed	
☐	☒	The exact sample size (*n*) for each experimental group/condition, given as a discrete number and unit of measurement
☐	☒	A statement on whether measurements were taken from distinct samples or whether the same sample was measured repeatedly
☐	☒	The statistical test(s) used AND whether they are one- or two-sided<br>*Only common tests should be described solely by name; describe more complex techniques in the Methods section.*
☒	☐	A description of all covariates tested
☐	☒	A description of any assumptions or corrections, such as tests of normality and adjustment for multiple comparisons
☐	☒	A full description of the statistical parameters including central tendency (e.g. means) or other basic estimates (e.g. regression coefficient) AND variation (e.g. standard deviation) or associated estimates of uncertainty (e.g. confidence intervals)
☒	☐	For null hypothesis testing, the test statistic (e.g. *F*, *t*, *r*) with confidence intervals, effect sizes, degrees of freedom and *P* value noted<br>*Give P values as exact values whenever suitable.*
☒	☐	For Bayesian analysis, information on the choice of priors and Markov chain Monte Carlo settings
☐	☒	For hierarchical and complex designs, identification of the appropriate level for tests and full reporting of outcomes
☐	☒	Estimates of effect sizes (e.g. Cohen's *d*, Pearson's *r*), indicating how they were calculated

Our web collection on statistics for biologists contains articles on many of the points above.

Software and code

Policy information about availability of computer code

Data collection	no software was used for data collection (experimental data only).
Data analysis	All scripts used to generate the paper results and Figures are provided on github (https://github.com/Kwon-DRFZ/Lupus).

For manuscripts utilizing custom algorithms or software that are central to the research but not yet described in published literature, software must be made available to editors and reviewers. We strongly encourage code deposition in a community repository (e.g. GitHub). See the Nature Portfolio guidelines for submitting code & software for further information.

Data

Policy information about availability of data

All manuscripts must include a data availability statement. This statement should provide the following information, where applicable:

- Accession codes, unique identifiers, or web links for publicly available datasets
- A description of any restrictions on data availability
- For clinical datasets or third party data, please ensure that the statement adheres to our policy

RNA-sequence and processed matrix data have been deposited in the ArrayExpress database at EMBL-EBI (www.ebi.ac.uk/arrayexpress) under accession number E-MTAB-12203.

Research involving human participants, their data, or biological material

Policy information about studies with human participants or human data. See also policy information about sex, gender (identity/presentation), and sexual orientation and race, ethnicity and racism.

Reporting on sex and gender	Patients of the investigated groups are mostly female, this is reflected in the frequency of female participants (~90%). In the group of healthy participants, the frequency of females was 83% (provided in Suppl. Table 1).
Reporting on race, ethnicity, or other socially relevant groupings	Patient recruitment was done according to the patients coming to the outpatient clinic at Charité Universitätsmedizin Berlin on a daily basis. Thus, all included patients were of caucasian background.
Population characteristics	Patient demographics are provided in Suppl. Table 1.
Recruitment	Participants were recruited based on inclusion criteria provided in Suppl. Table 1
Ethics oversight	The study was approved by the local ethics committee of Charité Universitätsmedizin Berlin

Note that full information on the approval of the study protocol must also be provided in the manuscript.

Field-specific reporting

Please select the one below that is the best fit for your research. If you are not sure, read the appropriate sections before making your selection.

☒ Life sciences ☐ Behavioural & social sciences ☐ Ecological, evolutionary & environmental sciences

For a reference copy of the document with all sections, see nature.com/documents/nr-reporting-summary-flat.pdf

Life sciences study design

All studies must disclose on these points even when the disclosure is negative.

Sample size	25 SLE, 23 pSS, and 24 HD samples were collected from patients.
Data exclusions	described in the method, 'Data processing procedures'
Replication	*Describe the measures taken to verify the reproducibility of the experimental findings. If all attempts at replication were successful, confirm this OR if there are any findings that were not replicated or cannot be reproduced, note this and describe why.*
Randomization	*Describe how samples/organisms/participants were allocated into experimental groups. If allocation was not random, describe how covariates were controlled OR if this is not relevant to your study, explain why.*
Blinding	*Describe whether the investigators were blinded to group allocation during data collection and/or analysis. If blinding was not possible, describe why OR explain why blinding was not relevant to your study.*

Reporting for specific materials, systems and methods

We require information from authors about some types of materials, experimental systems and methods used in many studies. Here, indicate whether each material, system or method listed is relevant to your study. If you are not sure if a list item applies to your research, read the appropriate section before selecting a response.

Materials & experimental systems

n/a	Involved in the study	
☐	☒	Antibodies
☒	☐	Eukaryotic cell lines
☒	☐	Palaeontology and archaeology
☒	☐	Animals and other organisms
☒	☐	Clinical data
☒	☐	Dual use research of concern
☒	☐	Plants

Methods

n/a	Involved in the study	
☒	☐	ChIP-seq
☐	☒	Flow cytometry
☒	☐	MRI-based neuroimaging

Antibodies

Antibodies used	All antibodies used are provided in the materials and methods section
Validation	Antibodies have been validated by distributors

Flow Cytometry

Plots

Confirm that:

☒ The axis labels state the marker and fluorochrome used (e.g. CD4-FITC).

☒ The axis scales are clearly visible. Include numbers along axes only for bottom left plot of group (a 'group' is an analysis of identical markers).

☒ All plots are contour plots with outliers or pseudocolor plots.

☒ A numerical value for number of cells or percentage (with statistics) is provided.

Methodology

Sample preparation	Cells were isolated by Ficoll and stained for sorting afterwards
Instrument	BD FACS Aria I or II
Software	BD FACS Diva, BD FlowJo
Cell population abundance	Purity checks were done after sorting, purity was above 90%
Gating strategy	A detailed gating strategy can be found in the supplemental material. Lymphocytes and single cells were identified based on their scatter properties and dead cells as well as CD14+ monocytes were excluded. B cells were gated by CD19 expression, NK cells by lack of CD3 and CD19 and positive staining for CD16 and CD7. T cells were identified by the lack of CD14, CD16, CD19 and positive expression of CD3 and were further subdivided into CD4+ and CD8+ cells. At baseline, conventional CD127 +CD25- cells (Tcon) in both T cell subsets were sorted.

☒ Tick this box to confirm that a figure exemplifying the gating strategy is provided in the Supplementary Information.
